# Whole Genome Characterization and Pathogenicity of a SC2020-1-Like PRRSV-1 Strain Emerging in Southwest China

**DOI:** 10.1155/2024/5627927

**Published:** 2024-10-15

**Authors:** Yuan-Meng Wang, Li-Shuang Deng, Bing-Zhou Huang, Han-Yu Li, Jia-Qi Duan, Yi-Xin Yan, Si-Yuan Lai, Yan-Ru Ai, Yuan-Cheng Zhou, Yi Qing, Zhi-Wen Xu, Ling Zhu

**Affiliations:** ^1^College of Veterinary Medicine, Sichuan Agricultural University, Chengdu 611130, China; ^2^Key Laboratory of Animal Breeding and Genetics Key Laboratory of Sichuan Province, Sichuan Animal Science Academy, Chengdu 611130, China; ^3^Sichuan Animal, Livestock and Poultry Biological Products Key Laboratory of Sichuan Province, Chengdu, China; ^4^Chengdu Livestock and Poultry Genetic Resources Protection Center, Chengdu 610081, China; ^5^Key Laboratory of Animal Diseases and Human Health of Sichuan Province, Chengdu 611130, China

## Abstract

Porcine reproductive and respiratory syndrome virus (PRRSV), encompassing PRRSV-1 and PRRSV-2, significantly impacts the global pig industry by causing reproductive disorders and respiratory difficulties. In this paper, we isolated a novel PRRSV-1 strain, named SCPJ2023, from weaned piglets in Sichuan. Utilizing primary macrophages, we isolated SCPJ2023 and performed complete genome sequencing through metagenomic analysis. Phylogenetic analysis classified SCPJ2023 as pan-European subtype 1. SCPJ2023 showed a 95.3% similarity to SC2020-1. Amino acid analysis identified differences in Nsp2, GP3, and GP4 between SCPJ2023 and other representative strains. In vivo challenge experiments demonstrated that SCPJ2023 induced clinical symptoms in piglets, including coughing, fever, reduced appetite, and depression. Pathological examinations revealed hemorrhage and congestion, increased inflammatory cells, thickening of the alveolar wall, and collapse of the alveolar cavity in SCPJ2023-infected piglets. Altogether, our study identified a novel pathogenic isolate of PRRSV-1, expanding the newly named SC2020-1-like subgroup by identifying additional strains beyond the initial SC2020-1 isolate.

## 1. Introduction

Porcine reproductive and respiratory syndrome (PRRS) is a devastating disease that results in reproductive failure in sows and respiratory problems in pigs of all ages, leading to significant economic repercussions worldwide [1]. Since it was initially reported in 1996, PRRS has been spreading within mainland China for over 20 years [2]. PRRS virus (PRRSV) is an enveloped, positive-sense, single-stranded RNA virus belonging to the *Arteriviridae* family, *Nidovirales* order. PRRSV is classified into two distinct species based on genetic and antigenic diversity: *Betaarterivirus suid* 1 (PRRSV-1) and *Betaarterivirus suid* 2 (PRRSV-2), with approximately 60% nucleotide identity at the genome-wide level [3, 4]. PRRSV-2 has garnered attention in China due to its rapid spread and severe pathogenicity, while PRRSV-1 has been quietly circulating in China for over two decades, establishing its own epidemic system [5–7]. Therefore, the threat posed by PRRSV-1 should not be underestimated.

The PRRSV genome is approximately 15 kb in length and consists of 5′-capped and 3′-polyadenylated regions with untranslated regions at the ends of both the 5′ and 3′ regions. It contains at least 11 open reading frames (ORFs), including ORF1a, ORF1b, ORF2a, ORF2b, ORF5a, and ORF3 to ORF7, as well as ORF2TF [8, 9]. A variety of structural proteins such as GP2, GP3, GP4, GP5a, GP5, M and N are encoded by ORFs 2-7, which are integral components of the viral structure. ORF1a and ORF1b encode the encoding of the viral nonstructural proteins (Nsps), which are critical for viral replication and immune response modulation [10]. Within the Nsps, the highly variable region (HVR) of the Nsp2-related proteins is characterized by frequent amino acid deletions or insertions, which are associated with diverse biological properties of the virus [11]. GP5 includes a signal peptide and two ectodomains, impacting the number of potential N-glycosylation sites. These features are pivotal for virus replication and the pig's antibody response [12]. Furthermore, the hypervariable regions within GP3 and GP4 of PRRSV-1 isolates are also noteworthy, as deletions frequently occur in these areas [13–15].

PRRSV-1 is primarily found in Eastern Europe and exhibits three, potentially four, subtypes [16]. Subtype 1 is primarily prevalent in Western and Central Europe but has been identified in many Asian countries, while the remaining subtypes are commonly recognized as the “Eastern European subtypes” [17]. The first PRRSV-1 detected in China was in 1997, with traces of the virus identified by Chinese customs during quarantine (B13, GenBank: AY633973) [18]. Subsequently, Chen et al. [19] isolated BJEU06 and NMEU09 strains in China for the first time in 2011 and analyzed their whole genome sequences. Due to the weak pathogenicity of most PRRSV-1 found in China, there were no studies on the pathogenicity of PRRSV-1 in China until 2016 [7, 20]. Most of the PRRSV-1 strains reported in China belonged to pan-European subtype 1, with common strains falling into four subgroups: BJEU06-like, NMEU09-like, Amervac-like, and HKEU16-like [6, 21]. Sun et al. [6] showed that the BJEU06-like strain was the most common PRRSV-1 strain, accounting for 45.2% of PRRSV-1 strains in China.

Although studies on PRRSV-1 have been conducted across most regions of China, pathogenicity analyses of PRRSV-1 isolates remain scarce, with the majority of these isolates classified under the BJEU06-like subgroup [22]. In this research, a PRRSV-1 strain (designated SCPJ2023, GenBank accession number: PP800763) from the lungs and blood of weaned piglets originating from a pig farm in Southwest China was discovered. We sequenced the complete genome of SCPJ2023 by metagenomic sequencing and analyzed its pathogenicity and genetic variation. Specifically, we introduce a new subgroup, including SC2020-1 and SCPJ2023, named SC2020-1-like, for the first time.

## 2. Materials and Methods

### 2.1. Sample Processing and Information

A total of 12 lung tissue and serum samples, confirmed as clinically positive for PRRSV, were obtained from pigs at swine farms where vaccination against PRRSV had not been implemented (Table 1). A large number of weaned piglets on the farm exhibited respiratory symptoms and decreased growth performance and appetite, with some piglets showing fever. Detection was performed using an unpublished real-time quantitative PCR (RT-qPCR) assay for PRRSV-1 developed in our laboratory (Table 2). The tissue samples were homogenized in PBS, and the supernatant was subsequently centrifuged to facilitate nucleic acid extraction. Total RNA was isolated using Trizol reagent (Takara, Dalian, China), following the manufacturer's instructions, and stored at −80°C.

### 2.2. Virus Isolation and Immunofuorescence Assay (IFA)

We employed Marc-145 cells and primary porcine alveolar macrophages (PAMs) for virus isolation. Marc-145 cells were sourced from our laboratory. The procurement of primary PAMs adhered to the method previously described [4]. Positive tissue samples underwent grinding and centrifugation, followed by filtration using 0.22 μm membranes. The filtrate was then inoculated onto both Marc-145 cells and primary PAMs for an incubation period of 1.5 h. Afterward, DMEM supplemented with 2% fetal bovine serum (FBS; Gibco, New York, USA) was added to the Marc-145 cells, while RPMI-1640 medium with the same concentration of FBS was used for the PAMs. The cells were incubated for 3 days at 37°C in 5% CO_2_ and continuously monitored for cytopathic effects (CPE). To detect SCPJ2023 replication in cells, PRRSV N protein-specific monoclonal antibodies (GeneTex, Carlsbad, USA) and fluorescein isothiocyanate-conjugated secondary antibodies (Proteintech, Rosemont, USA) were used for indirect IFAs. The cycle threshold (Ct) values of cell culture were detected by RT-qPCR. Subsequently, the isolated viruses were carefully collected and stored at −80°C.

### 2.3. Complete Genome Sequencing and Genetic Analysis

RNA from PAMs infected with SCPJ2023 was submitted to Tanpu Biotechnology Co., Ltd. (Shanghai, China) for metagenomic sequencing as described previously [23]. To investigate the evolutionary relationships between the newly identified isolates and other strains of PRRSV, we performed sequence analysis using DNASTAR software (version 7.1). The multiple sequence alignments included 51 selected representative strains (51 PRRSV-1 and 3 PRRSV-2) obtained from the GenBank database. A phylogenetic analysis was performed based on these alignments using MEGA 7.0 software, employing 1000 bootstrap replicates for each node. The tree was constructed using the neighbor-joining method, known for its effectiveness in illustrating evolutionary relationships [24]. Additionally, the iTOL online platform was utilized to annotate the phylogenetic tree (https://itol.embl.de/) [25].

### 2.4. Amino Acid Mutation and Recombination Analysis

The characteristics of amino acid mutations were analyzed by means of comparing the amino acid sequences of PRRSV GP3, GP4, GP5, and Nsp2 genes utilizing the Clustal W module embedded in DNASTAR software. To analyze the recombination events of SCPJ2023, we applied multiple bioinformatics tools and methods. First, we downloaded the complete genome sequences of SCPJ2023, SC2020-1, and a variety of representative PRRSV strains from the GenBank database. Then, we used the MEGA software for sequence alignment to ensure high quality and consistency. After alignment, we used the RDP4 (Recombination Detection Program 4) software to carry on the preliminary restructuring event detection. RDP4 includes seven different detection methods, including RDP, GENECONV, Bootscan, MaxChi, Chimaera, SiScan, and 3Seq, to improve the reliability and accuracy of detection. The significance of each method set is to *p*  < 0.05. To validate the preliminary results, we also used SimPlot software for rechecks, which also identified potential reconfiguration hotspots and reconfiguration breakpoints.

### 2.5. Animals and Experimental Design

In this study, 10 2-week-old piglets were selected, all confirmed to be free of antibodies and antigens against several viruses, including PRRSV, pseudorabies virus, African swine fever virus, classical swine fever virus, porcine circovirus 2, and porcine circovirus 3. Piglets were randomly divided into two groups. The experimental group consisted of piglets intranasally challenged with 2 ml of SCPJ2023 (10^5.0^TCID_50_/ml). The control group was intranasally inoculated with 2 ml of RPMI 1640 medium. The rectal temperature, body weight, survival rate, and clinical manifestations of piglets in each group were evaluated daily. Serum samples were collected at predetermined intervals on 1, 3, 5, 7, 9, 11, and 14 days postinoculation (dpi). All surviving piglets were humanely euthanized and subjected to a comprehensive postmortem examination at 14 dpi. Tissue samples were collected for virus detection, histopathological analysis, and immunohistochemical examination, as previously described [26, 27]. The samples were fixed in 10% neutral buffered formalin and processed for H&E staining and immunohistochemistry. For immunohistochemical analysis, a PRRSV N protein-specific monoclonal antibody (GeneTex, Carlsbad, USA) was used as the primary antibody.

### 2.6. Data Analysis

GraphPad 8.0 was utilized for the organization and analysis of experimental data, which encompassed body temperature, body weight, and viral load of the animals. Significant differences were determined using *t*-tests and nonparametric tests in GraphPad 8.0 (San Diego, CA, USA), with a significance level set at *p*  < 0.05.

### 2.7. Ethical Statement

The experimental procedures involving animals in this study were ethically approved by the Experimental Animal Management Committee of Sichuan Agricultural University, following established animal welfare guidelines (Approval No. SYXK2019-187).

## 3. Results

### 3.1. Metagenomic Sequencing of SCPJ2023 Genomic

**Figure 1:**
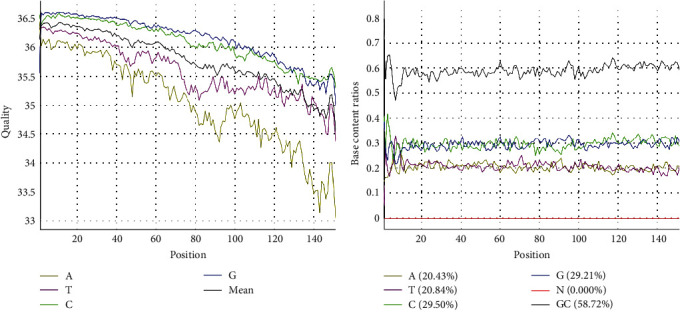
The base quality distribution of the raw data (A) indicated satisfactory base quality, while the base composition distribution (GC bias plot) of the raw data (B) showed a relatively stable base ratio, with the GC content within the expected range.

**Figure 2:**
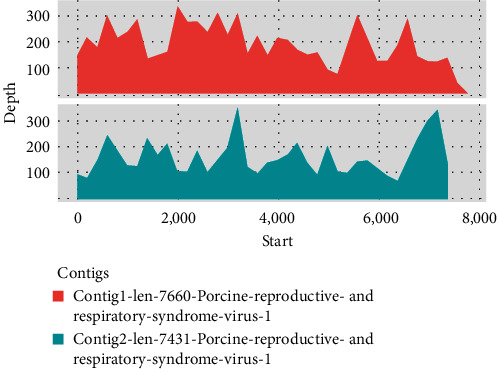
The average sequencing depths for Contig1 and Contig2 were 195 and 160, respectively. After assembly, the resulting sequence showed the highest similarity to the SC2020-1 sequence.

The complete genome of the SCPJ2023 isolate was determined by metagenomic sequencing. After filtering, the GC content of the reads was found to be 58.72% (Figure 1). Quality tests showed that the filtered Q20 and Q30 rates were 97.94% and 94.09%, respectively, indicating satisfactory data quality (Supporting Information 1: Table S1). After removing ribosomal RNA and bacterial sequences, two sequences with lengths of 7660 and 7431 bp were generated (Figure 2). BLAST analysis revealed that these sequences had the highest similarity with SC2020-1, with similarities of 96.031% and 94.563%, respectively. The final sequence was assembled to obtain a complete sequence of 15,043 bp.

### 3.2. PRRSV-1 SCPJ2023 Isolated in PAMs

**Figure 3:**
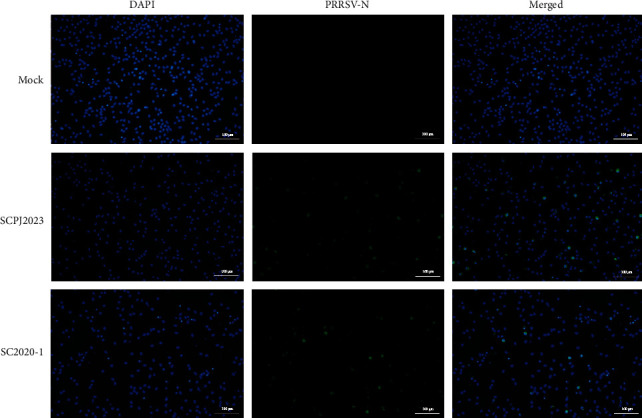
The IFA results for SC2020-1 and SCPJ2023. The results showed that reactivity could be detected in SCPJ2023-infected PAMs, which was consistent with the results of SC2020-1-infected PAMs (positive control). There was no fluorescence signal in the negative control group. Scale bar = 100 μm.

**Figure 4:**
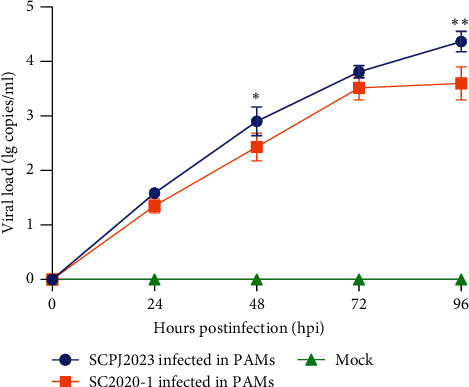
Growth kinetics of SCPJ2023and SC2020-1. The growth curves showed that the in vitro replication rate of SCPJ2023 isolated in PAMs was higher than that of SC2020-1 isolated (*p*  < 0.05). Significant differences are marked with asterisks.  ^*∗∗∗∗*^*p*  < 0.0001;  ^*∗∗∗*^*p*  < 0.001;  ^*∗∗*^*p*  < 0.01;  ^*∗*^*p*  < 0.05.

SCPJ2023 was blindly passaged for three generations in Marc-145 cells and PAMs. The results showed no CPE in Marc-145 cells, and no Ct value was detected in the third generation by RT-qPCR, indicating that SCPJ2023 could not replicate in Marc-145 cells. However, CPE was observed in SCPJ2023 cultured in PAMs after 72 h, and the Ct value disappeared in the fourth generation by RT-qPCR. The growth dynamic curves of SCPJ2023 and SC2020-1 showed that the replication efficiency of SCPJ2023 was higher than that of SC2020-1 isolates (Figure 3). The results of immunofluorescence detection showed that the PAMs cells infected with SCPJ2023 and SC2020-1 exhibited obvious green fluorescence signals, but no fluorescence signal was detected in the uninfected control cells (Figure 4). These results confirm the successful isolation of SCPJ2023 in PAMs.

### 3.3. Genomic Character of SCPJ2023

**Figure 5:**
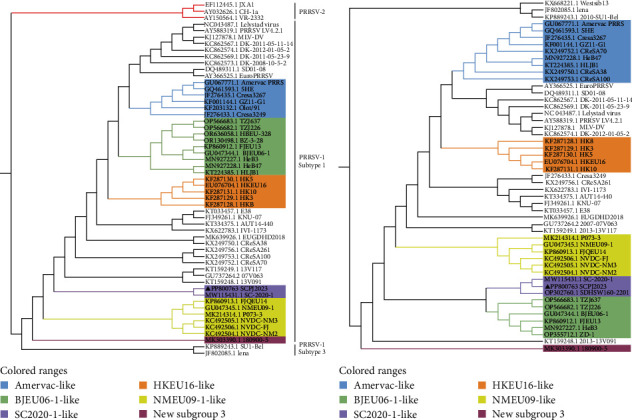
Phylogenetic analysis of 53 typical PRRSV-1 strains based on the whole genome (A), phylogenetic analysis based on ORF5 (B). SCPJ2023 isolates clustered with SC2020-1 isolated. Each strain was denoted by its GenBank accession number and strain name. Subgroups of Chinese PRRSV-1 isolates were shown in different colors. The isolate in this study was marked with black triangles (▲).

The complete genomic length of SCPJ2023 was determined to be 15,043 nucleotides (nt) (GenBank accession number: PP800763), which is classified within the PRRSV-1 group. The nucleotide identities of SCPJ2023 with the representative PRRSV-1 strain (Lelystad virus, LV) and the representative PRRSV-2 strain (ATCC VR-2332) were 86.58% and 60.5%, respectively. The phylogenetic tree based on representative sequences downloaded from the GenBank database (51 strains of PRRSV-1 and 3 strains of PRRSV-2) indicated that SCPJ2023 belonged to subtype 1 of PRRSV-1 (Supporting Information 2: Table S2). The SCPJ2023 isolate, which clusters with SC2020-1 and shares 95.3% sequence similarity with it, has been identified as belonging to a new subgroup previously described by Sun et al. [6] (Figure 5). In this paper, we designate this new subgroup as “SC2020-1 like.”

### 3.4. Amino Acid Mutation and Recombination Analysis

**Figure 6:**
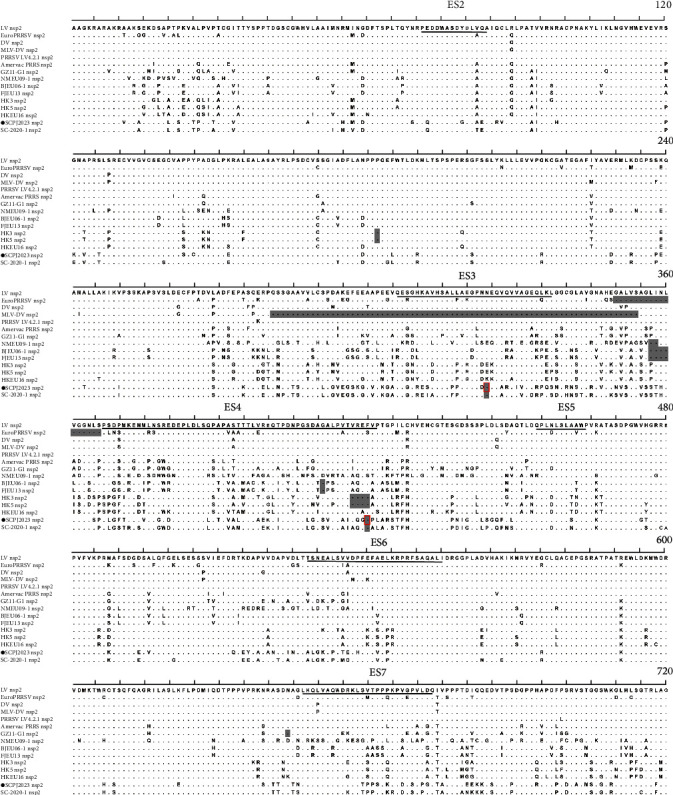
Alignment of the partial Nsp2 amino acid sequence between the SCPJ2023 isolate and representative Chinese PRRSV-1 strains. All of the sequences were marked with gray shading, and discontinuities in the isolate were marked with solid red boxes. Previously identified B-cell epitopes (ES) were represented by black underscores. The isolate in this study was marked with black circles (●).

Nsp2, the largest multidomain protein in PRRSV, plays a crucial role in protein processing and regulation of the immune response during viral replication. The amino acid homology between the SCPJ2023 strain and the representative strain Lelystad in the Nsp2 region is 80.7% (Table 3). Nsp2 has been characterized by six B-cell epitopes, among which ES2 is situated within the putative cysteine domain crucial for viral survival and regulation of immune response [28]. SCPJ2023 exhibited four amino acid mutations in the ES2 region compared to the representative strain Lelystad and one amino acid substitution relative to SC2020-1. The hypervariable regions ES3 and ES4 of Nsp2 frequently exhibit significant variability. Similar to SC2020-1, SCPJ2023 also displayed a 2-amino acid (1 + 1) deletion indel at the 323 aa in the ES3 region and the 424 aa in the ES4 region, along with numerous amino acid substitution events in both regions (Figure 6).

**Figure 7:**
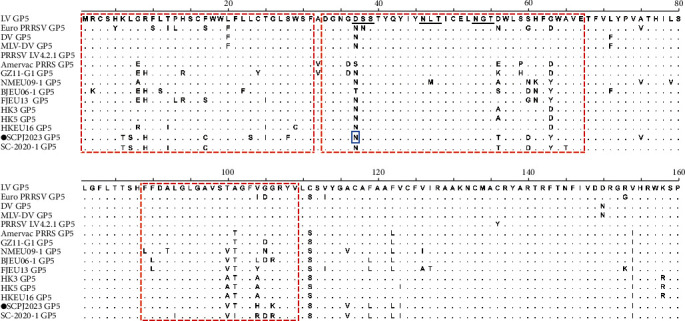
Alignment of the partial GP5 amino acid sequence between the isolated strain and representative Chinese PRRSV-1 strains. The assumed signal sequences were indicated by the first red dashed boxes, and the hypervariable domains were denoted by the remaining two red dashed boxes. Potential N-glycosylation sites were delineated by black underlining. The mutations of the SCPJ2023 isolate at location 37 (N37) were indicated using a solid blue wireframe. The strain isolated in this study was marked with black circles (●).

**Figure 8:**
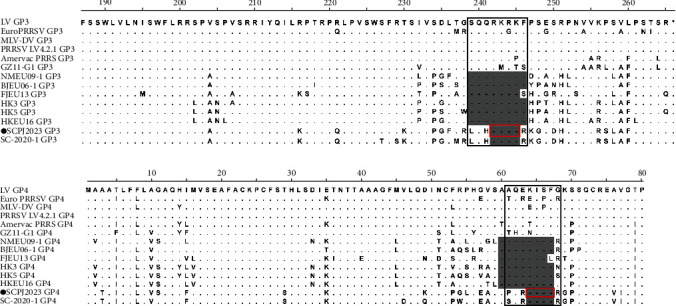
Alignment of amino acid sequences of GP3 and GP4 between the SCPJ2023 strain and representative Chinese PRRSV-1 strains. The amino acid deletions of GP3 and GP4 were marked with gray shading, and the deletions of the novel isolate were marked with an additional solid red wire frame. The overlap between GP3 and GP4 was marked with a solid black box. The stop codon was denoted by an asterisk ( ^*∗*^). The novel isolate in this study was shown in black circles (●).

GP5, an envelope glycoprotein consisting of 201 aa, is encoded by sequence ORF5 [29]. SCPJ2023 shows 15 aa substitutions in the hypervariable region, including 7 aa substitutions in the signal peptide region. Additionally, SCPJ2023 had a substitution at 37 aa, a potential glycosylation site (Figure 7). SCPJ2023 shows 4-aa deletion in the hypervariable region that overlaps GP3 and GP4, specifically at 242-245 aa of GP3 and 64-67 aa of GP4 (Figure 8).

Combined analysis of RDP4 and Simplot revealed no recombination events in SCPJ2023 (data not shown). Although SC2020-1 and SCPJ2023 nucleotide homology of 95%, rather than the genetic differences between them due to mutations restructuring events.

### 3.5. Pathogenicity Analysis

#### 3.5.1. Autopsy Lesions and Tissue Staining Analysis

**Figure 9:**
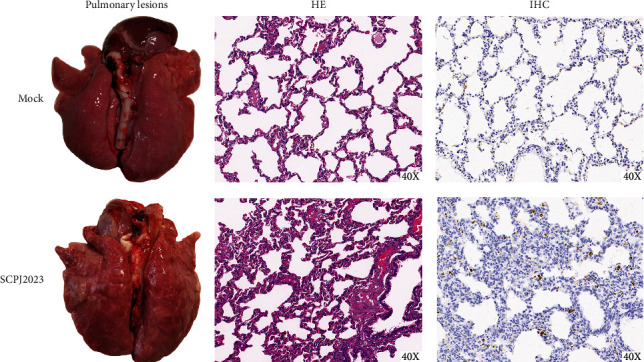
Gross lung lesions and histopathological examination of both the experimental and control groups. SCPJ2023-infected pigs excited pulmonary congestion, dull color, and hemorrhage. H&E results revealed hemorrhage and congestion, increased inflammatory cells, thickening of the alveolar wall, and collapse of the alveolar cavity in SCPJ2023-infected pigs. IHC showed that PRRSV-specific antigen could be detected.

To assess the pathogenicity of the SCPJ2023 isolate, a pig challenge experiment was conducted. During the trial, the SCPJ2023-infected group exhibited clinical symptoms, including cough, fever, decreased appetite, and depression. All piglets were humanely euthanized at 14 dpi. Pulmonary lesions in the two experimental groups varied in severity (Figure 9). Tissues were collected for hematoxylin and eosin (H&E) staining. The histological analysis revealed that the lungs of pigs challenged with SCPJ2023 exhibited hemorrhage and congestion, increased inflammatory cells, thickening of the alveolar wall, and collapse of the alveolar cavity. No significant abnormalities were observed in the control group. IHC results showed that an obvious brown staining signal was observed in the lung sections infected with SCPJ2023, mainly concentrated in alveolar epithelial cells. In contrast, no specific brown staining signal was detected in uninfected control lung sections. These results indicated that SCPJ2023 successfully infected the lungs.

#### 3.5.2. Recording and Analysis of Data From Pig Challenge Experiments

**Figure 10:**
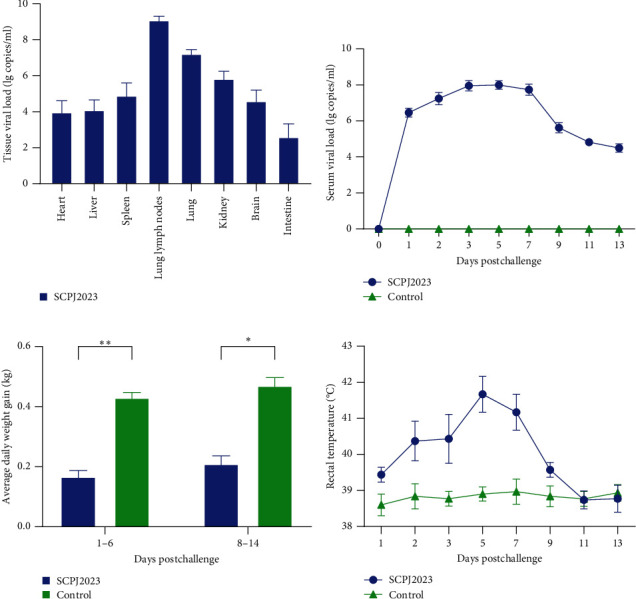
The results of rectal temperature, weight gain, kinetics of viremia, and viral load in each organ during the challenge experiments. (A) The highest viral loads were consistently found in the lungs and lung lymph nodes. (B) SCPJ2023 infections resulted in reduced weight gain in piglets. (C) Viremia commenced within 24 h in piglets challenged with SCPJ2023, reaching peak levels on 3 dpi. (D) SCPJ2023 challenges induced fever (above 40°C) in piglets from 2 to 7 dpi. Significant differences are marked with asterisks and “ns”:  ^*∗∗∗∗*^*p* < 0.0001;  ^*∗∗∗*^*p* < 0.001;  ^*∗∗*^*p* < 0.01;  ^*∗*^*p* < 0.05.

The experimental group exhibited a fever (rectal temperature exceeding 40°C) starting from 2 dpi, persisting for 5 days and peaking at 5 dpi. Viremia was detected in experimental groups 24 h after the challenge, reaching its peak at 5 dpi. During the trial, it was observed that the experimental group exhibited lower weight gain compared to the control group (Figure 10), and the difference with the control group was significant (*p* < 0.05). Viral loads were detected in all tissues of the experimental animals upon dissection, with the highest concentrations observed in the lungs and lung lymph nodes.

## 4. Discussion

PRRSV causes reproductive disorders and respiratory difficulties in pigs. First isolated in the United States in 1987, PRRSV has since spread globally, inflicting substantial economic losses on the global pig industry [30]. PRRSV has two genotypes, PRRSV-1 and PRRSV-2, and they have coexisted in China for more than 20 years [31]. Initial isolations of wild-type PRRSV-1 strains in China, BJEU06-1 and NMEU09-1, were reported in 2011 [18]. But Chinese customs had intercepted PRRSV-1 infected pigs before (B13, GenBank: AY633973), suggesting that PRRSV-1 could have been introduced earlier than thought. Since then, PRRSV-1 has been increasingly detected in over 20 provinces, including Henan and Shandong [19, 32–34]. In our study, the phylogenetic analysis results identified SCPJ2023 isolate represents a new subgroup, designated as SC2020-1-like, along with SC2020-1. The ORF5 phylogenetic analysis revealed the presence of an additional strain, SDHSW160-2201, within the SC2020-1 subgroup. However, only the ORF5 sequences are available for this strain, limiting further investigation. This finding suggests that the SC2020-1 subgroup may be more widespread than previously recognized. Our research not only isolates a new strain of PRRSV-1 but also discusses its pathogenicity and genomic features, offering valuable insights for further research into PRRSV-1. Additionally, we introduce a new subgroup, including SC2020-1 and SCPJ2023, named SC2020-1-like, for the first time.

The sequence homology analysis revealed that SCPJ2023 and SC2020-1 exhibited less than 90% sequence similarity with other representative strains but shared a 95.3% sequence similarity with each other. Furthermore, both strains were isolated from the same province (not the same farm). It is highly probable that SCPJ2023 has evolved from SC2020-1. This observation suggests that SC2020-1 underwent rapid mutation, giving rise to new strains within a span of just 3 years.

Most of the pan-European subtype 1 strains are medium and low virulence strains. Studies on the pathogenicity of PRRSV-1 in China were scarce, with only six reports (Table 4). Among these, three strains were classified as Amervac-like (HLJB1, GZ11-G1, and NPUST 2789-3W-2), while two were identified as BJEU06-like (ZD-1 and SD1291) [7, 22, 36–38]. The remaining strain was associated with an Italian strain in a distinct subgroup (181187-2) [38]. Notably, none of these six strains resulted in pig mortality. However, an increasing number of highly pathogenic PRRSV-1 strains have been identified in international studies. The mortality rate of the NV 2022 strain was 23.8% [7]. Infections with the westsib13 strain have resulted in symptoms including anorexia, dyspnea, and tremors in piglets, with a mortality rate reaching 100% [39]. Therefore, continued monitoring of PRRSV-1 is essential. Our research demonstrated that while this particular strain was not lethal, it did provoke symptoms including cough, fever, decreased appetite, and depression in piglets. Although many isolates of PRRSV-1 are present in China, there is still a scarcity of studies on their pathogenicity. This study contributes to a deeper understanding of the pathogenic characteristics of PRRSV-1.

Nsp2 is the most variable functional protein in PRRSV, playing a crucial role in viral assembly, recombination, immune regulation, cell apoptosis, cell cycle, and cell morphology [19, 37, 40–42]. It is cleaved from ORF1 translation products to form mature proteins, consisting of HV-I, PLP2, HV-2, TM, and C-terminal domains, exhibiting significant sequence heterogeneity across different strains [43]. In Chinese PRRSV-1 genomes, the most common deletion indel involves deletions at 357–360 aa + 411 aa [6, 14]. However, Han et al. [11] showed that deletion of this part did not affect viral replication. In this study, the SCPJ2023 isolate exhibited a deletion indel distinct from the common indel but identical to the SC2020-1 isolate, specifically a 2-aa (1 + 1) deletion at 323 aa and 424 aa. Research indicates that deletions in Nsp2 predominantly occur in the B-cell epitope zone of its HVR, which may act as a decoy antigen epitope to disrupt host immune responses [11]. There were a large number of amino acid mutation events in ES2 and hypervariable regions (ES3 and ES4). In addition, amino acid deletion events occurred in ES3 (323aa) and ES4 (424 aa).

To further analyze the structural proteins of this isolate, the GP3, GP4, and GP5 proteins were compared with representative strains. Amino acid comparisons based on GP5 revealed that SCPJ2023 isolate exhibited substitutions in the HVR and mutations at the potential glycosylation site (37 aa). Mutations in GP5 typically occur in the outer domain, which contains numerous potential N-glycosylation sites [12]. These sites are critical for viral immune escape and antibody neutralization [44]. Overall, the GP5 protein of the SCPJ2023 isolate demonstrated considerable conservation. Among the structural proteins of PRRSV-1, GP3 and GP4 are the most variable. They share an overlapping hypervariable region corresponding to the 237–252 aa region of GP3 and the 57–72 aa region of GP4 [28]. The HVR of GP4, which overlaps with GP3 as identified in earlier studies, may function as a decoy epitope and is closely associated with dispersed immune responses [45]. Most PRRSV-1 isolates in China typically exhibit amino acid deletions in this region [15]. Similar deletion events were also observed in the SCPJ2023 isolate (242–245 aa of GP3 and 64–67 aa of GP4). The similarity in deletion patterns suggests that these isolates may share a common or closely related ancestry, potentially aiding their adaptation [46]. SCPJ2023 and SC2020-1 shared identical characteristics in the HVR of GP3 and GP4, providing further evidence that SCPJ2023 may have evolved from SC2020-1. This deletion pattern was not reported in the remaining PRRSV-1 isolates, including different isolates from China and other countries, providing new insights into the genomic diversity and evolution of PRRSV-1. Current reported PRRSV-1 isolates, such as Lena, WestSib13, TZJ226, TZJ637, and ZD-1, all exhibit premature termination at the C-terminal end of GP3 [33, 37, 39, 47]. Although this feature was not observed in our study, the prevalence of premature termination in China and its potential impact on virulence warrants further investigation.

In conclusion, we successfully isolated a new strain of PRRSV-1 from weaned piglets, providing insights into its genomic characteristics and pathogenicity. Phylogenetic analysis based on ORF5 and the whole genome indicated that SCPJ2023 is a new member of the SC2020-1-like subgroup belonging to subtype 1. Pathogenicity analysis revealed that SCPJ2023-infected piglets exhibited hemorrhage, congestion, increased infiltration of inflammatory cells, alveolar wall thickening, and alveolar collapse. Our study provides valuable data on PRRSV-1 and emphasizes the necessity of continuous surveillance of its evolution and dissemination in China.

## Figures and Tables

**Table 1:** Clinical sample information used for PRRSV1 detection.

Sample no.	Specimen	*C*_T_^a^	Judgment (+/−)^b^	Province	Positive rate
No. 1	Lung	27.68	+	Sichuan	75%
	Serum	29.34	+		
No. 2	Lung	23.73	+		
	Serum	24.98	+		
No. 3	Lung	22.74	+		
	Serum	21.89	+		
No. 4	Lung	38.66	−		
	Serum	Undetermined	−		
No. 5	Lung	29.15	+		
	Serum	30.15	+		
No. 6	Lung	20.88	+		
	Serum	22.59	+		
No. 7	Lung	34.62	−		
	Serum	Undetermined	−		
No. 8	Lung	28.56	+		
	Serum	30.44	+		

^a^ *C*_T_ < 33 is considered test positive, *C*_T_ ≥ 33 is considered test negative.

^b^The symbol “+” indicates a positive test result, and “−” indicates a negative test result.

**Table 2:** Primer information of RT-qPCR.

Primer	Sequence information	Tm (°C)
Forward primer	TCCATTCAATCCCAGCGTC	55
Reverse primer	CGAAGTCCTGGTACTAGAGTG	
Probe	ATACGCYSTKAGAAAGCCC	—

**Table 3:** Comprehensive genomic and amino acid homology comparison of SCPJ2023 isolate with representative PRRSV-1 strains.

Nucleotides region	Similarity to SCPJ2023 (%)					
	Lelystad virus (%)	Amervac PRRS (%)	BJEU06-1 (%)	HKEU16 (%)	NMEU09-1 (%)	SC-2020-1 (%)
ORF1a	84.5	83.3	82.0	81.6	79.4	94.5
ORF1b	88.8	87.9	86.0	86.4	85.2	96.0
ORF2a	88.0	86.4	85.6	86.3	86.3	95.2
ORF2b	93.4	93.0	94.4	89.7	92.0	96.2
ORF3	85.5	85.0	84.4	86.6	86.2	95.5
ORF4	85.0	85.2	83.9	84.6	85.2	97.0
ORF5	86.1	86.1	86.5	84.3	84.5	95.0
ORF6	89.7	90.2	87.2	88.9	91.0	97.7
ORF7	93.0	91.5	89.9	88.9	90.7	96.1
Complete	86.6	85.6	84.2	84.1	82.9	95.3
Protein						
Nsp1	84.7	84.1	81.6	79.6	80.9	94.4
Nsp2	80.7	78.6	78.0	77.8	73.3	92.9
Nsp3	86.6	85.9	84.2	84.6	83.1	95.6
Nsp4	86.4	87.7	83.9	84.1	82.1	86.2
Nsp5	88.4	85.9	85.7	86.9	81.8	82.0
Nsp6	83.3	83.3	77.1	79.2	81.2	97.9
Nsp7	88.6	87.4	87.0	85.3	85.3	95.7
Nsp8	89.6	84.4	89.6	87.4	88.9	98.5
Nsp9	89.7	88.9	86.7	87.1	85.5	96.1
Nsp10	87.0	86.3	85.1	84.5	83.8	95.9
Nsp11	90.0	87.4	85.7	87.6	85.7	97.2
Nsp12	89.3	89.3	86.2	86.8	86.8	94.1
GP2	90.4	87.2	88.8	88.4	86.4	95.6
E	94.4	93.0	93.0	91.5	95.8	97.2
GP3	83.2	82.4	82.9	83.7	86.8	96.6
GP4	82.8	83.9	85.2	84.7	85.2	95.0
GP5	86.6	87.6	87.6	88.1	87.1	93.6
M	94.3	93.7	89.7	92.0	93.7	97.1
N	94.6	93.8	87.6	88.4	93.8	96.1

**Table 4:** Comparison of pathogenicity of SCPJ2023 strains and other strains in China.

Infected PRRSV strain	Subtype	Region	Clinical signs and symptoms	Pathological and histopathological lesions	Growth inhibition	Reference
SC2020-1	Subtype 1	Chengdu Mianyang, Sichuan, China	Abortion in sows and respiratory disorders in piglets	No data	No data	[35]

SCPJ2023	Subtype 1	Chengdu, Sichuan, China	Fever (≥40°C), medium clinical symptoms	Hemorrhage and congestion, increased inflammatory cells, thickening of alveolar wall, and collapse of alveolar cavity.	Significantly inhibit growth	This study

NPUST-2789-3 W-2	Subtype 1	China Taiwan	No fever, mild clinical symptoms	Diffuse and severe interstitial pneumonia.	No significant	[36]

ZD-1	Subtype 1	China Heilongjiang	Fever (≥40°C), medium clinical symptoms	Consolidation in the lungs, extensive infammatory cell infltration with alveolar epithelial proliferation and moderate alveolar diaphragm widening in the lungs.	significantly inhibit growth in 8-14 days	[37]

HLJB1	Subtype 1	China Heilongjiang	Fever (≥40°C), mild clinical symptoms	Pulmonary consolidation and septal thickening with red blood cell infiltration.	No data	[20]

GZ11-G1	Subtype 1	China Guizhou	Fever (≥40°C), mild clinical symptoms	Interstitial pneumonia with severely alveolar septa thickening, and alveoli disappearance and atrophy, and many macrophages and necrotic debris within the bronchiole and peribronchiolitis.	No data	[7]

181187-2	Subtype 1	China Henan	Fever (≥40°C), mild clinical symptoms	Interstitial pneumonia, inflammatory cell infiltration.	Significantly inhibit growth	[38]

SD1291	Subtype 1	China Shandong	Fever (≥40°C), mild clinical symptoms	Interstitial pneumonia with infltration of lymphocytes and widened alveolar septum.	Significantly inhibit growth	[22]

## Data Availability

The genome sequences identified in this study have been deposited in the GenBank database. Raw data can be obtained from the corresponding author upon reasonable request.

## Supporting Information

Supporting Information 1 Table S1: metagenomics sequencing quality.

Supporting Information 2 Table S2: Genbank accession number of the representative strains.

## References

[1] Nathues H., Alarcon P., Rushton J. (2017). Cost of Porcine Reproductive and Respiratory Syndrome Virus at Individual Farm Level—An Economic Disease Model. *Preventive Veterinary Medicine*.

[2] Zimmerman J. J., Dee S. A., Holtkamp D. J. (2019). Porcine Reproductive and Respiratory Syndrome Viruses (Porcine Arteriviruses). *Diseases of Swine*.

[3] Collins J. E., Benfield D. A., Christianson W. T. (1992). Isolation of Swine Infertility and Respiratory Syndrome Virus (Isolate Atcc Vr-2332) in North America and Experimental Reproduction of the Disease in Gnotobiotic Pigs. *Journal of Veterinary Diagnostic Investigation*.

[4] Wensvoort G., Terpstra C., Pol J. M. (1991). Mystery Swine Disease in the Netherlands: The Isolation of Lelystad Virus. *Veterinary Quarterly*.

[5] Valicek L., Psikal I., Smid B., Rodak L., Kubalikova R., Kosinova E. (1997). Isolation and Identification of Porcine Reproductive and Respiratory Syndrome Virus in Cell Cultures. *Veterinarni Medicina*.

[6] Sun Q., Xu H., An T., Cai X., Tian Z., Zhang H. (2023). Recent Progress in Studies of Porcine Reproductive and Respiratory Syndrome Virus 1 in China. *Viruses*.

[7] Wang X., Yang X., Zhou R. (2016). Genomic Characterization and Pathogenicity of a Strain of Type 1 Porcine Reproductive and Respiratory Syndrome Virus. *Virus Research*.

[8] Lunney J. K., Fang Y., Ladinig A. (2016). Porcine Reproductive and Respiratory Syndrome Virus (Prrsv): Pathogenesis and Interaction with the Immune System. *Annual Review of Animal Biosciences*.

[9] Cui X., Xia D., Huang X. (2022). Analysis of Recombinant Characteristics Based on 949 Prrsv-2 Genomic Sequences Obtained From 1991 to 2021 Shows that Viral Multiplication Ability Contributes to Dominant Recombination. *Microbiology Spectrum*.

[10] Bautista E. M., Faaberg K. S., Mickelson D., McGruder E. D. (2002). Functional Properties of the Predicted Helicase of Porcine Reproductive and Respiratory Syndrome Virus. *Virology*.

[11] Han J., Liu G., Wang Y., Faaberg K. S. (2007). Identification of Nonessential Regions of the nsp2 Replicase Protein of Porcine Reproductive and Respiratory Syndrome Virus Strain VR-2332 for Replication in Cell Culture. *Journal of Virology*.

[12] Dea S., Gagnon C. A., Mardassi H., Pirzadeh B., Rogan D. (2000). Current Knowledge On the Structural Proteins of Porcine Reproductive and Respiratory Syndrome (Prrs) Virus: Comparison of the North American and European Isolates. *Archives of Virology*.

[13] Zhou Z., Liu Q., Hu D. (2015). Complete Genomic Characterization and Genetic Diversity of Four European Genotype Porcine Reproductive and Respiratory Syndrome Virus Isolates From China in 2011. *Virus Genes*.

[14] Zhang Q., Song Z., Yu Y., Huang J., Jiang P., Shan H. (2020). Genetic Analysis of a Porcine Reproductive and Respiratory Syndrome Virus 1 Strain in China with New Patterns of Amino Acid Deletions in Nsp2, Gp3 and Gp4. *Microbial Pathogenesis*.

[15] Lee D. U., Yoo S. J., Kwon T. (2017). Genetic Diversity of Orf 4-6 of Type 1 Porcine Reproductive and Respiratory Syndrome Virus in Naturally Infected Pigs. *Veterinary Microbiology*.

[16] Morgan S. B., Graham S. P., Salguero F. J. (2013). Increased Pathogenicity of European Porcine Reproductive and Respiratory Syndrome Virus Is Associated with Enhanced Adaptive Responses and Viral Clearance. *Veterinary Microbiology*.

[17] Stadejek T., Oleksiewicz M. B., Scherbakov A. V. (2008). Definition of Subtypes in the European Genotype of Porcine Reproductive and Respiratory Syndrome Virus: Nucleocapsid Characteristics and Geographical Distribution in Europe. *Archives of Virology*.

[18] Yun Z., Zjixiong L., Chen R. (1998). Molecular Cloning and Identification of the Orf_(7) Gene of Chinese Isolates B_(13) of Porcine Reproductive and Respiratory Syndrome Virus.

[19] Chen N., Cao Z., Yu X. (2011). Emergence of Novel European Genotype Porcine Reproductive and Respiratory Syndrome Virus in Mainland China. *Journal of General Virology*.

[20] Ming S., Yongying M., Bohua L. (2017). Pathogenic Characterization of European Genotype Porcine Reproductive and Respiratory Syndrome Virus Recently Isolated in Mainland China. *The Open Virology Journal*.

[21] Chen N., Liu Q., Qiao M., Deng X., Chen X., Sun M. (2017). Whole Genome Characterization of a Novel Porcine Reproductive and Respiratory Syndrome Virus 1 Isolate: Genetic Evidence for Recombination Between Amervac Vaccine and Circulating Strains in Mainland China. *Infection, Genetics and Evolution*.

[22] Li C., Qiu M., Li S. (2023). Metagenomic and Pathogenic Assessments Identify a Pathogenic Porcine Reproductive and Respiratory Syndrome Virus 1 With New Deletions From Adult Slaughter Pig in 2022. *Transboundary and Emerging Diseases*.

[23] Qiu M., Li S., Xiao Y. (2022). Pathogenic and Metagenomic Evaluations Reveal the Correlations of Porcine Epidemic Diarrhea Virus, Porcine Kobuvirus and Porcine Astroviruses With Neonatal Piglet Diarrhea. *Microbial Pathogenesis*.

[24] Kumar S., Stecher G., Tamura K. (2016). MEGA7: Molecular Evolutionary Genetics Analysis Version 7.0 for Bigger Datasets. *Molecular Biology and Evolution*.

[25] Letunic I., Bork P. (2021). Interactive Tree Of Life (iTOL) v5: An Online Tool for Phylogenetic Tree Display and Annotation. *Nucleic Acids Research*.

[26] Chen N., Li S., Ye M. (2018). A Novel Nadc30-Like Porcine Reproductive and Respiratory Syndrome Virus (PRRSV) Plays a Limited Role in the Pathogenicity of Porcine Circoviruses (PCV2 and PCV3) and PRRSV Co-Infection. *Transboundary and Emerging Diseases*.

[27] Feldman A. T., Wolfe D. (2014). Tissue Processing and Hematoxylin and Eosin Staining. *Methods Mol Biology*.

[28] Ropp S. L., Wees C. E., Fang Y. (2004). Characterization of Emerging European-Like Porcine Reproductive and Respiratory Syndrome Virus Isolates in the United States. *Journal of Virology*.

[29] Shi M., Lam T. T., Hon C. C. (2010). Molecular Epidemiology of PRRSV: A Phylogenetic Perspective. *Virus Research*.

[30] Allende R., Lewis T. L., Lu Z. (1999). North American and European Porcine Reproductive and Respiratory Syndrome Viruses Differ in Non-Structural Protein Coding Regions. *Journal of General Virology*.

[31] Han K., Seo H. W., Park C. (2013). Comparative Pathogenicity of Three Korean and One Lelystad Type 1 Porcine Reproductive and Respiratory Syndrome Virus (Pan-European Subtype 1) Isolates in Experimentally Infected Pigs. *Journal of Comparative Pathology*.

[32] Chen N., Xiao Y., Ye M. (2020). High Genetic Diversity of Chinese Porcine Reproductive and Respiratory Syndrome Viruses From 2016 to 2019. *Research in Veterinary Science*.

[33] Li C., Xu H., Zhao J. (2022). Epidemiological Investigation and Genetic Evolutionary Analysis of PRRSV-1 on a Pig Farm in China. *Frontiers in Microbiology*.

[34] Gao J. C., Xiong J. Y., Ye C. (2017). Genotypic and Geographical Distribution of Porcine Reproductive and Respiratory Syndrome Viruses in Mainland China in 1996–2016. *Veterinary Microbiology*.

[35] Zhao J., Zhu L., Deng H. (2021). Genetic Characterization of a Novel Porcine Reproductive and Respiratory Syndrome Virus Type I Strain From Southwest China. *Archives of Virology*.

[36] Hsueh F.-C., Kuo K.-L., Hsu F.-Y. (2023). Molecular Characteristics and Pathogenicity of Porcine Reproductive and Respiratory Syndrome Virus (Prrsv) 1 in Taiwan During 2019–2020. *Life*.

[37] Xu H., Gong B., Sun Q. (2023). Genomic Characterization and Pathogenicity of Bjeu06-1-Like PRRSV-1 Zd-1 Isolated in China. *Transboundary and Emerging Diseases*.

[38] Wang X., Bai X., Wang Y. (2023). Pathogenicity Characterization of PRRSV-1 181187-2 Isolated in China. *Microbial Pathogenesis*.

[39] Yuzhakov A. G., Raev S. A., Skrylev A. N. (2017). Genetic and Pathogenic Characterization of a Russian Subtype 2 PRRSV-1 Isolate. *Veterinary Microbiology*.

[40] Yu F., Liu L., Tian X. (2022). Genomic Analysis of Porcine Reproductive and Respiratory Syndrome Virus 1 Revealed Extensive Recombination and Potential Introduction Events in China. *Veterinary Sciences*.

[41] Morgan S. B., Frossard J. P., Pallares F. J. (2016). Pathology and Virus Distribution in the Lung and Lymphoid Tissues of Pigs Experimentally Inoculated with Three Distinct Type 1 PRRS Virus Isolates of Varying Pathogenicity. *Transboundary and Emerging Diseases*.

[42] Sinn L. J., Klingler E., Lamp B. (2016). Emergence of a Virulent Porcine Reproductive and Respiratory Syndrome Virus (PRRSV) 1 Strain in Lower Austria. *Porcine Health Management*.

[43] Shen S., Kwang J., Liu W., Liu D. X. (2000). Determination of the Complete Nucleotide Sequence of a Vaccine Strain of Porcine Reproductive and Respiratory Syndrome Virus and Identification of the Nsp2 Gene with a Unique Insertion. *Archives of Virology*.

[44] Gonin P., Pirzadeh B., Gagnon C. A., Dea S. (1999). Seroneutralization of Porcine Reproductive and Respiratory Syndrome Virus Correlates with Antibody Response to the Gp5 Major Envelope Glycoprotein. *Journal of Veterinary Diagnostic Investigation*.

[45] Costers S., Vanhee M., Van Breedam W., Van Doorsselaere J., Geldhof M., Nauwynck H. J. (2010). Gp4-Specific Neutralizing Antibodies Might be a Driving Force in PRRSV Evolution. *Virus Research*.

[46] Drew T. W., Lowings J. P., Yapp F. (1997). Variation in Open Reading Frames 3, 4 and 7 Among Porcine Reproductive and Respiratory Syndrome Virus Isolates in the UK. *Veterinary Microbiology*.

[47] Karniychuk U. U., Geldhof M., Vanhee M., Van Doorsselaere J., Saveleva T. A., Nauwynck H. J. (2010). Pathogenesis and Antigenic Characterization of a New East European Subtype 3 Porcine Reproductive and Respiratory Syndrome Virus Isolate. *BMC Veterinary Research*.

