# Supplementary material for: Whole Genome Characterization and Pathogenicity of a SC2020-1-Like PRRSV-1 Strain Emerging in Southwest China
Source: Transbound Emerg Dis. 2024 Oct 15;2024:5627927. doi: 10.1155/2024/5627927 (PMC12016694; doi:10.1155/2024/5627927)
Supplement: Supporting Information 1 — Table S1: metagenomics sequencing quality. [file 5627927.f1.docx]

| QC | Before filtering | After filtering |
| --- | --- | --- |
| Total reads | 191790060 | 166873616 |
| Total bases | 28960299060 | 19939825449 |
| Q20 bases | 27731863087 | 19528631609 |
| Q30 bases | 26318016964 | 18761064840 |
| Q20 rate | 95.76% | 97.94% |
| GC content | 59.84% | 58.72% |

Supplementary table 1. Metagenomics sequencing quality.
