# Supplementary material for: Whole Genome Characterization and Pathogenicity of a SC2020-1-Like PRRSV-1 Strain Emerging in Southwest China
Source: Transbound Emerg Dis. 2024 Oct 15;2024:5627927. doi: 10.1155/2024/5627927 (PMC12016694; doi:10.1155/2024/5627927)
Supplement: Supporting Information 2 — Table S2: Genbank accession number of the representative strains. [file 5627927.f2.docx]

| No. | Virus Strain | Accession No. | No. | Virus Strain | Accession No. |
| --- | --- | --- | --- | --- | --- |
| 1 | Amervac PRRS | GU067771.1 | 28 | EUGDHD2018 | [MK639926.1](https://www.ncbi.nlm.nih.gov/nuccore/MK639926.1) |
| 2 | Lelystad virus | NC_043487.1 | 29 | 180900-5 | [MK303390.1](https://www.ncbi.nlm.nih.gov/nuccore/MK303390.1) |
| 3 | lena | JF802085.1 | 30 | FJQEU14 | [KP860913.1](https://www.ncbi.nlm.nih.gov/nuccore/KP860913.1) |
| 4 | MLV-DV | KJ127878.1 | 31 | NMEU09-1 | [GU047345.1](https://www.ncbi.nlm.nih.gov/nuccore/GU047345.1) |
| 5 | PRRSV LV4.2.1 | [AY588319.1](https://www.ncbi.nlm.nih.gov/nuccore/AY588319.) | 32 | NVDC-FJ | [KC492506.1](https://www.ncbi.nlm.nih.gov/nuccore/KC492506.1) |
| 6 | SHE | [GQ461593.1](https://www.ncbi.nlm.nih.gov/nuccore/GQ461593.1) | 33 | NVDC-NM2 | [KC492504.1](https://www.ncbi.nlm.nih.gov/nuccore/KC492504.1) |
| 7 | TZJ637 | [OP566683.1](https://www.ncbi.nlm.nih.gov/nuccore/OP566683.1) | 34 | NVDC-NM3 | [KC492505.1](https://www.ncbi.nlm.nih.gov/nuccore/KC492505.1) |
| 8 | HBEU-328 | [OR636058.1](https://www.ncbi.nlm.nih.gov/nuccore/OR636058.1) | 35 | 2013-13V091-Belgium | [KT159248.1](https://www.ncbi.nlm.nih.gov/nuccore/952985705) |
| 9 | DK-2011-05-23-9 | [KC862569.1](https://www.ncbi.nlm.nih.gov/nuccore/KC862569.1) | 36 | 2007-07V063-Belgium | [GU737264.2](https://www.ncbi.nlm.nih.gov/nuccore/339409383) |
| 10 | EuroPRRSV | [AY366525.1](https://www.ncbi.nlm.nih.gov/nuccore/38146324) | 37 | 2013-13V117-Belgium | [KT159249.1](https://www.ncbi.nlm.nih.gov/nuccore/952985716) |
| 11 | DK-2011-05-11-14 | [KC862567.1](https://www.ncbi.nlm.nih.gov/nuccore/KC862567.1) | 38 | 2010-SU1-Belarus | [KP889243.1](https://www.ncbi.nlm.nih.gov/nuccore/KP889243.1) |
| 12 | DK-2008-10-5-2 | [KC862573.1](https://www.ncbi.nlm.nih.gov/nuccore/KC862573.1) | 39 | SD01-08 | [DQ489311.1](https://www.ncbi.nlm.nih.gov/nuccore/DQ489311) |
| 13 | FJ0603 | [HM114313.1](https://www.ncbi.nlm.nih.gov/nuccore/HM114313.1) | 40 | KNU-07 | [FJ349261.1](https://www.ncbi.nlm.nih.gov/nuccore/FJ349261) |
| 14 | GZ11-G1 | [KF001144.1](https://www.ncbi.nlm.nih.gov/nuccore/KF001144.1) | 41 | HLJB1 | [KT224385.1](https://www.ncbi.nlm.nih.gov/nuccore/KT224385.1) |
| 15 | HeB47 | [MN927228.1](https://www.ncbi.nlm.nih.gov/nuccore/MN927228.1) | 42 | AUT14-440 | [KT334375.1](https://www.ncbi.nlm.nih.gov/nuccore/KT334375) |
| 16 | BJEU06-1 | [GU047344.1](https://www.ncbi.nlm.nih.gov/nuccore/GU047344.1) | 43 | IVI-1173 | [KX622783.1](https://www.ncbi.nlm.nih.gov/nuccore/KX622783) |
| 17 | FJEU13 | [KP860912.1](https://www.ncbi.nlm.nih.gov/nuccore/KP860912.1) | 44 | Olot/91 | [KF203132.1](https://www.ncbi.nlm.nih.gov/nuccore/KF203132) |
| 18 | HeB3 | [MN927227.1](https://www.ncbi.nlm.nih.gov/nuccore/MN927227.1) | 45 | CReSA261 | [KX249756.1](https://www.ncbi.nlm.nih.gov/nuccore/KX249756.1) |
| 19 | NVDC-NM1-2011 | [JX187609.1](https://www.ncbi.nlm.nih.gov/nuccore/JX187609.1) | 46 | CReSA38 | [KX249750.1](https://www.ncbi.nlm.nih.gov/nuccore/KX249750.1) |
| 20 | TZJ226 | [OP566682.1](https://www.ncbi.nlm.nih.gov/nuccore/OP566682.1) | 47 | Cresa3249 | [JF276433.1](https://www.ncbi.nlm.nih.gov/nuccore/JF276433.1) |
| 21 | CReSA70 | [KX249752.1](https://www.ncbi.nlm.nih.gov/nuccore/KX249752) | 48 | Cresa3267 | [JF276435.1](https://www.ncbi.nlm.nih.gov/nuccore/JF276435.1) |
| 22 | HK3 | [KF287129.1](https://www.ncbi.nlm.nih.gov/nuccore/KF287129.1) | 49 | E38 | [KT033457.1](https://www.ncbi.nlm.nih.gov/nuccore/KT033457.1?report=genbank&to=15065) |
| 23 | HK5 | [KF287130.1](https://www.ncbi.nlm.nih.gov/nuccore/KF287130.1) | 50 | CReSA100 | [KX249753.1](https://www.ncbi.nlm.nih.gov/nuccore/KX249753.1) |
| 24 | HK8 | [KF287128.1](https://www.ncbi.nlm.nih.gov/nuccore/KF287128.1) | 51 | DK-2012-01-05-2 | [KC862574.1](https://www.ncbi.nlm.nih.gov/nuccore/KC862574.1) |
| 25 | HK10 | [KF287131.1](https://www.ncbi.nlm.nih.gov/nuccore/KF287131.1) | 52 | VR-2332 | AY150564.1 |
| 26 | HKEU16 | [EU076704.1](https://www.ncbi.nlm.nih.gov/nuccore/EU076704.1) | 53 | JXA1 | EF112445.1 |
| 27 | SC-2020-1 | [MW115431.1](https://www.ncbi.nlm.nih.gov/nuccore/MW115431.1) | 54 | CH-1a | AY032626.1 |

Supplementary table 2: Genbank accession number of the representative strains
